# Mental distress, food insecurity and university student dropout during the COVID-19 pandemic in 2020: evidence from South Africa

**DOI:** 10.3389/fpsyt.2024.1336538

**Published:** 2024-02-06

**Authors:** Fezile Wagner, Ryan G. Wagner, Lerato P. Makuapane, Mxolisi Masango, Unathi Kolanisi, Francesc Xavier Gómez-Olivé

**Affiliations:** ^1^Analytics and Institutional Research Unit (AIRU), University of the Witwatersrand, Johannesburg, South Africa; ^2^MRC/Wits Rural Public Health and Health Transitions Research Unit (Agincourt), School of Public Health, Faculty of Health Sciences, University of the Witwatersrand, Johannesburg, South Africa; ^3^Department of Institutional Planning (DIP), University of Pretoria, Pretoria, South Africa; ^4^Department of Consumer Sciences, Faculty of Science, Agriculture and Engineering, University of Zululand, KwaDlangezwa, South Africa

**Keywords:** universities, college, attrition, depression, anxiety, Africa

## Abstract

**Background:**

Student dropout has been a key issue facing universities for many years. The COVID-19 pandemic was expected to exacerbate these trends; however, international literature has produced conflicting findings. Limited literature from Africa has investigated the impact of COVID-19 on student dropout trends, despite the documented devastation, including increased risk of food insecurity and mental distress, caused by the pandemic.

**Objective:**

This work seeks to understand the impact of food insecurity and mental distress on student dropout during the COVID-19 pandemic.

**Methods:**

Using a cross-sectional research design, first-year undergraduate students from a large South African university were recruited via email to participate in a survey between September and October 2020. The Household Food Insecurity Access Scale (HFIAS) was used to measure food insecurity and the Patient Health Questionnaire Anxiety and Depression Scale (PHQ-ADS) was used to measure mental distress. Multivariate regression was used to investigate factors associated with student dropout.

**Results:**

The student dropout rate was 10.5% (95% CI: 8.2-13.2). The prevalence of severe food insecurity was 25.7% (95% CI: 22.3-29.4) and the prevalence of severe mental distress symptoms was 26.7% (95% CI: 23.3-30.4). Dropout rates and levels of food insecurity were highest among students residing in remote areas during the lockdown at 19.2% and 43.6%, respectively. The multivariate logistic regression revealed that being male increased the probability of dropout almost three-fold (odds ratio (OR) = 2.70; 95% CI: 1.48-4.89, p =0.001)). Being moderately food insecure increased the odds of dropout more than two-fold (OR=2.50; 95% CI:1.12-5.55, p=0.025), and experiencing severe mental distress symptoms increased the odds of dropout seven-fold (OR=7.08; 95% CI:2.67-18.81, p<0.001).

**Conclusion:**

While acknowledging that various factors and complexities contribute to student dropout, the increased vulnerability to food insecurity and mental distress, stemming from issues such as widespread job losses and isolation experienced during the pandemic, may have also had an impact on dropout. This work reiterates the importance of directing additional support to students who are food insecure and those who are experiencing mental distress in order to mitigate university student dropout.

## 1 Introduction

University student dropout and retention rates are commonly used in higher education to describe the enrolment status of students following admission into an academic programme (1). Student dropout, or attrition, captures the decline in the number of students initially enrolled in an academic programme, while retention represents the count of students who continue to re-enroll in the program in subsequent years until completion (1, 2).

Considerable efforts have focused on monitoring and understanding student dropout rates, particularly in the first year of study which typically has the highest dropout rates ranging from 8-21% (3, 4). A comprehensive review of empirical literature classifies dropout determinants into five categories (4): i. *student demographic factors-* with being a male student increasing dropout probability (5), ii. *family background*- with lower socio-economic status increasing the likelihood of dropout (6), iii. *academic and social integration*- with greater ties to peers and institutional commitment reducing dropout rates (7), iv. *institutional factors*- with large class sizes increasing dropout rates (8) and v. *labour market trends* – with employability prospects having mixed impact on student dropout. The contribution of psychosocial and wellbeing variables, such as mental health and food security, on student dropout trends have generally been underexplored.

Recently, there has been general concern about the negative impact of COVID-19 on student dropout. A study from Europe reported a significant increase in dropout rates, especially among students with children and disabilities (9). A study from South America reported higher levels of dropout, citing economic and mental challenges related to the pandemic as key factors (1). In South Africa, preliminary analysis conducted using national data from the South African Department of Higher Education (DHET) early in the pandemic indicated increased dropout rates when compared to the previous years, with a significantly larger increase of dropout among those not receiving financial aid (2.8%) (10). While these findings cannot be solely attributed to factors related to the pandemic, they do justify further exploration of the factors that might have influenced student dropout during the pandemic.

South African higher education’s response to the COVID-19 pandemic forced students to move back home to environments that were often unconducive for learning (11, 12). Students were expected to adopt a new and complex mode of learning which required computer hardware and connectivity (12, 13)). Many students faced realities of job and income loss (3, 13, 14). They were also anxious about contracting COVID-19, with some falling ill, some having to be primary caregivers, and others grieving for those who had passed away (3).

Several studies have reported on the impact of COVID-19 on students’ food security (13–15). Literature has identified job loss as a key contributor to student food insecurity during the pandemic (13, 16, 17). Studies also noted increased levels of mental distress among university students, triggered by the move to online learning, poor home environments, financial concerns as well as anxiousness precipitating from isolation and confinement due to lockdown (11, 18).

Preceding the onset of the COVID-19 pandemic, scholarly investigations unveiled robust connections between food insecurity, mental distress, and student progression (19, 20). The pandemic and subsequent lockdown heightened these risks by i) amplifying the threat of food insecurity through loss of livelihoods, ii) inducing mental distress through experiences of grief, isolation, and uncertainty among students, and iii) ushering in a novel mode of remote teaching and learning that imposed constraints on certain student groups. It is expected that the interplay of these factors would negatively influence student dropout rates. The current study, therefore, aims to understand the impact of food insecurity and mental distress on university student dropout during the COVID-19 pandemic in South Africa.

This study is informed by Tinto’s interactionalist theory on student departure (7). Social and academic integration are critical elements in Tinto’s theory. Academic integration is conceptualised as intra-curricular interactions between students and university staff, as well as their peers. Social interaction is defined as the extent to which students feel connected to and involved in the social life of the university community. Tinto posits that a student’s choice to discontinue their university enrolment unfolds through a complex series of experiences. Tinto’s theory acknowledges the role of both socio-economic factors as well as factors contributing to isolation rather than integration, as critical factors influencing student departure decisions. Drawing from Tinto’s theory, the confluence of financial strain (and resulting impact of food insecurity), coupled with mental distress, some of which emanated from feelings of isolation, despondency and loneliness during the COVID-19 pandemic, created a precarious environment for students, potentially leading to a decision to dropout.

## 2 Materials and methods

### 2.1 Study context

The research was conducted at an urban South African university with ~41,000 students enrolled in 2020. The student body was majority female (55%) with most students (60%) enrolled for undergraduate studies. Among those enrolled, the largest population group was Black, accounting for 61% of all students. Generally, the student population represented all population groups and all official languages of South Africa.

In March 2020, the university suspended all contact activities and advised students to vacate university residences following the declaration of a nationwide state of disaster by the South African government in response to the COVID-19 pandemic (21). The South African lockdown was characterized by a 5-level alert system aimed at managing the spread of the virus (22). Alert level 5 was the most stringent and mandated residence confinement. Alert level 1, the least strict, was implemented during periods of low COVID-19 transmission (22). During the lockdown, the university’s academic programme continued online. The national state of disaster was officially lifted on April 5, 2022 (23).

### 2.2 Sample

The current research forms part of a larger, cross-sectional survey that sought to understand the impact of COVID-19 on the student population. The primary focus of the present analysis is on a specific group of students where dropout has traditionally been highest, namely first year students. As such, the inclusion criteria were: first-time entering, first-year students who were enrolled in full-time undergraduate programmes, aged 18 years and older and had biographical information on the university’s system. Students who did not meet these criteria were not included in the analysis.

### 2.3 Data collection

Data collection occurred between September and October 2020, coinciding with South Africa’s COVID-19 lockdown alert levels 2 and 1. After obtaining ethical clearance and approval from the university registrar, a list of email addresses belonging to individuals who met the inclusion criteria was compiled. Recruitment for the study began by sending out emails to these individuals. Upon agreeing through an online consent process, participants proceeded to complete a self-administered online survey hosted on the Research Electronic Data Capture (REDCap) platform (24). The 2021 registration status of all study participants was used to assess dropout.

### 2.4 Variables and measures

#### 2.4.1 Food insecurity

The Household Food Insecurity Access Scale (HFIAS) was administered in English and used to assess food insecurity among students. The HFIAS consists of nine items, with responses to each item captured in one of three categories: i. rarely (once or twice in the past four weeks), ii. sometimes (three to ten times in the past four weeks), and iii. often (more than ten times in the past four weeks). The HFIAS utilizes an algorithm that classifies food security status into four categories: food secure, mildly food insecure, moderately food insecure and severely food insecure. These categories have been used in similar studies (20, 25).

The HFIAS is one of the most predominantly used tools to measure food insecurity among South African university students (20, 25–27). The HFIAS tool has been found to have satisfactory reproducibility and validity in studies among university students. Validation studies among university students reported good internal consistencies, with Cronbach α values ranging from 0.920 in Germany, 0.750 in Lebanon, and 0.916 among South African university students (26, 28, 29). The current study yielded a good internal consistency of 0.950.

#### 2.4.2 Mental distress

The Patient Health Questionnaire Anxiety and Depression Scale (PHQ-ADS), a composite scale, was used to measure mental distress. The PHQ-ADS combines the sum scores of the PHQ-9 as well as the Generalized Anxiety Disorder-7 (GAD-7). The PHQ-9 is a self-report questionnaire containing nine-items and requires participants to reflect on several depressive symptoms. The GAD-7, also a self-report questionnaire, contains seven items used to screen for anxiety symptoms. Both the PHQ-9 and GAD-7 use a two-week recall period. They were administered in English with responses captured in four categories i. not at all, ii. several days, iii. more than half the days, and iv. nearly every day. The PHQ-ADS, a combination of the PHQ-9 and GAD-7 tools, has a scale from 0-48, with cutoffs: 0–10, denoting minimal mental distress; 11–20, denoting mild mental distress; 21–30, denoting moderate mental distress; and 31–48, denoting severe mental distress. These cutoffs have been used in similar studies (30, 31).

The PHQ-9 has been used extensively in university settings both in South Africa, and other parts of the world (32–35). Validation studies have found the PHQ-9 to have good construct validity and reliability. Validation studies among university students reported internal consistencies of α=0.85 in Nigeria, α=0.83 in South Africa and α=0.84 in Iran (36–38). The PHQ-9 was also found to have good test-retest reliability (r=0.894, p<0.001) and displayed good convergence with the GAD-7 and PHQ-ADS at 0.751 and 0.934, respectively, and both significant at p<0.001 (31, 36b). The current study yielded a good internal consistency of 0.871.

The GAD-7 is regularly used to ascertain levels of generalised anxiety among students (32, 33; 35, 39). Validation studies have found the GAD-7 to have good construct validity and reliability. Validation studies among university students reported good internal consistencies, with Cronbach α values ≥ 0.85 in studies taking place in the United States of America, 0.903 among Spanish students and 0.892 among South African university students (32, 40, 41). The GAD-7 also displayed good convergent validity with the PHQ-9 and PHQ-ADS with coefficients ≥0.75 (30). The current study yielded a good internal consistency of 0.913.

#### 2.4.3 Student dropout

Using official university records, students participating in this research who were enrolled in 2020 but failed to re-enroll at any time in the year 2021 were defined as having dropped out. Students who re-enrolled were described as ‘retained’. As such, in the current study, dropout status was a binary variable reflecting: 1) those who dropped out or, 2) those who were retained. This definition of dropout is aligned with definitions found in earlier literature (2).

#### 2.4.4 Socio-demographic variables

Variables included in this research were: self-identified sex (male or female), population group (Black, White, Coloured, Indian and Chinese), first-generation status (yes or no) referring to individuals who were first in their family to attend university, whether participants were recipients of financial aid (yes or no), subject area participants were enrolled in (Commerce, Law & Management, Engineering, Health Sciences, Humanities and Sciences), as well as high school quintile (1-5 and other [‘other’ referring to participants who matriculated outside of the South African public system]). The school quintile variable is used in South Africa to classify public schools based on the socio-economic conditions of the communities they serve. Quintile 1 schools are found in low-resource areas, while quintile 5 schools are in the most affluent communities (42).

#### 2.4.5 COVID-19 and lockdown related variables

This research also aimed to capture factors relating to COVID-19 and the subsequent lockdown. The variable ‘Self-reported COVID-19 infection’ sought to understand if research participants and/or their close friends and family had ever been infected with the COVID-19 virus. Responses were categorized as ‘yes’ for those who had been infected and ‘no’ for those who had never been infected. Data on the location of the participant’s residence during lockdown was also captured and coded as ‘City/Suburb,’ ‘Township,’ ‘Town,’ or ‘Village/Farm’. The variable ‘income disruption’ captured whether household income during this period: ‘increased’, ‘decreased’, ‘remained the same’, or was ‘unknown’. The final three variables captured whether ‘working from home’, having ‘limited workspace at home’ as well as general ‘home circumstances’ were challenging during this time, to which responses were captured as either ‘yes’ or ‘no’.

### 2.5 Statistical analyses

The data underwent cleaning and analysis using STATA software (version 17; College Station, Texas, USA). Descriptive analyses were conducted on all variables, and proportions and 95% confidence intervals (95% CI) were reported, as appropriate. To compare categorical variables related to dropout, the chi-square test was utilized, while the Mann-Whitney U test was employed to compare the continuous age variable. To account for differences between the sample and the population, data were weighted based on sex and population group before calculating prevalence and constructing the logistic regression model. A forward and backward stepwise regression, with a inclusion cut-off of p-value ≤ 0.20 used to identify variables included in the final logistic regression model (43). Statistical significance was defined at a p-value ≤ 0.05 for all analyses.

## 3 Results

A total of 5,684 students fulfilled the study’s inclusion criteria and were invited to take part in this study. Of those invited, 12.8% (726) participated. Records with no data in the variables of interest were removed from the sample, forming an analytical sample of 596 (10.5%) of the entire student sample and 82% of those who participated).

### 3.1 Sample characteristics

The crude student dropout rate among study participants was 9.9% (95% CI: 7.7-12.6) and the weighted 10.5% (95% CI: 8.2-13.2). Significantly higher percentages of dropout were noted among male participants when compared to female participants (14.1% versus 7.7%, respectively; p=0.013). Black (11.0%) and White (10.9%) (**Table 1**) participants had the highest proportions of dropout. Dropout levels were lowest for students who attended quintile 5 high schools (9.7%) and schools falling in the ‘other’ category (8.0%), with students who dropped out being significantly older (p=0.005).

**Table 1:** Unweighted sample socio-demographic characteristics by dropout status.

	Total sample n=596	Dropout status		p-value
		Retained	Dropped out	
	% (n)	% (n)	% (n)	
**Sex**				0.013*
Female	65.5 (390)	92.3 (360)	7.7 (30)	
Male	34.6 (206)	85.9 (177)	14.1 (29)	
**Population group**				0.297
Black	61.4 (366)	89.1 (326)	11.0 (40)	
Chinese	0.50 (3)	100.0 (3)	0.0 (0)	
Coloured	5.70 (34)	100.0 (34)	0.0 (0)	
Indian	12.4 (74)	91.9 (68)	8.1 (6)	
White	20.0 (119)	89.1 (106)	10.9 (13)	
Age M(SD)**	20 (± 2.3)	19.8 (± 2.0)	20.1 (± 3.3)	0.005*
**High school quintile**				0.153
1 (Lowest resourced schools)	5.5 (33)	81.8 (27)	45.5 (15)	
2	9.4 (56)	81.2 (46)	37.5 (21)	
3	12.1 (72)	91.7 (66)	22.2 (16)	
4	11.1 (66)	89.4 (59)	18.2 (12)	
5 (Highest resourced schools)	34.6 (206)	91.8 (189)	9.7 (20)	
Other (International or private high schools)	98.8 (163)	92.0 (150)	8.0 (13)	
**Subject area**				0.714
Commerce, Law & Management	13.4 (81)	93.8 (76)	5 (6.2)	
Engineering	21.3 (127)	89.0 (113)	11.0 (14)	
Health Sciences	14.8 (88)	88.6 (78)	11.4 (10)	
Humanities	31.4 (187)	91.0 (170)	9.1 (17)	
Sciences	19.0 (113)	88.5 (100)	11.5 (13)	
**First-generation status**				0.897
First-generation	39.8 (237)	90.3 (214)	9.7 (23)	
Non-first generation	60.2 (359)	90.0 (323)	10.0 (36)	
**Financial-aid recipient**				0.734
Yes	44.5 (265)	90.6 (240)	9.4 (25)	
No	55.5 (331)	89.7 (297)	10.3 (34)	
**Self- reported disability Status**				0.329
Yes	3.0 (18)	83.3 (15)	16.7 (3)	
No	97.0 (578)	90.3 (533)	9.7 (56)	

*Significance at p<0.05 (p-values represent frequency diﬀerences between dropout and retention).

** Age described by median values, standard deviation, and the Mann-Whitney U test.

### 3.2 COVID-19 and lockdown factors impacting on wellbeing

A higher proportion of dropout (15.2%) was noted amongst participants who reported being infected with COVID-19 or knew of close friends and family members who had been infected; however, this association was not statistically significant (**Table 2**). Participants whose residence during the time of the survey was in a village or farm had a higher dropout rate (19.2%; p=0.041). In terms of the impact of COVID-19 and lockdown on income, those who reported an increase in income during this time reported proportionally lower levels of dropout (6.3%), compared to those reporting a decrease (10.1%) or no change in income (9.9%); this association was not significant.

**Table 2:** Unweighted COVID-19 and lockdown factors with an impact on participants’ wellbeing by dropout status.

	Total sample n=596	Dropout status		p-value
		Retained	Dropped out	
	% (n)	% (n)	% (n)	
**Self- reported COVID-19 infection (of participant and/or close friends and family)**				0.209
Yes	7.7 (46)	84.8 (39)	15.2 (7)	
No	92.3 (550)	90.5 (498)	9.5 (52)	
**Residence during lockdown situated in:**				0.041*
City/Suburb	60.0 (357)	91.0 (325)	9.0 (32)	
Township	20.3 (121)	92.6 (112)	7.4 (9)	
Town	7.4 (44)	91.0 (40)	9.1 (5)	
Village/farm	12.3 (73)	80.8 (59)	19.2 (14)	
**Income disruption**				0.879
Decrease	48.0 (286)	89.9 (257)	10.1 (29)	
Remain the same/unknown	49.3 (294)	90.1 (265)	9.9 (29)	
Increase	2.7 (16)	93.8 (15)	6.3 (1)	
**Working from home challenging**				0.836
Yes	6.9 (361)	90.3 (326)	9.7 (35)	
No	17.8 (235)	89.8 (211)	10.2 (24)	
**Home circumstances challenging**				0.624
Yes	52.2 (311)	90.7 (282)	9.3 (29)	
No	47.8 (285)	89.5 (255)	10.5 (30)	
**Limited workspace at home**				0.380
Yes	51.0 (301)	89.0 (268)	11.0 (33)	
No	50.0 (295)	91.2 (269)	8.8 (26)	

*Significance at p<0.05 (p-values represent frequency diﬀerences between dropout and retention).

### 3.3 Food insecurity and mental distress by dropout

The prevalence of severe food insecurity among participants was 25.7% (95% CI: 22.3-29.4) (**Table 3**). Students reporting food insecurity were significantly more likely to dropout (p=0.046). Furthermore, students living in a village or a farm during lockdown had significantly higher rates of severe (43.6%) and moderate food insecurity (32.8%) compared to students living in the city or suburbs (17.7% and 17.4%, respectively; p<0.001).

**Table 3:** Population-weighted food insecurity and mental health categories of study participants by dropout status.

	Prevalence	Dropout status		p value
		Retained	Dropped out	
	% (95% CI)	%	%	
**Food insecurity**				0.046*
Food secure	37.9 (95% CI: 34.1-41.9)	92.6%	7.4%	
Mildly food insecure	15.2 (95% CI: 12.6-18.4)	91.9%	8.1%	
Moderately food insecure	21.2 (95% CI: 18.7-24.7)	82.5%	17.5%	
Severely food insecure	25.7 (95% CI: 22.3-29.4)	89.3%	10.7%	
**Mental distress**				<0.001*
Minimal	18.2 (95% CI: 15.3- 21.5)	93.7%	6.3%	
Mild	30.7 (95% CI: 27.1- 34.5)	94.1%	5.9%	
Moderate	24.4 (95% CI: 21.2-28.1)	93.3%	6.7%	
Severe	26.7 (95% CI: 23.3-30.4)	78.0%	22.0%	

*Significance at p<0.05 (p-values represent frequency differences between dropout and retention); data presented as values weighted for sex and population group.

The prevalence of severe mental distress symptoms was 26.7% (95% CI: 23.3-30.4), with significant differences between the severity of mental distress symptoms and student dropout (p< 0.001), generally showing higher levels of dropout in students with greater depressive symptomology.

### 3.4 Factors associated with dropout

The multivariable regression model (**Table 4**) revealed that being male increased the probability of dropout almost three-fold (odds ratio [OR] = 2.70; 95% CI: 1.48-4.89; p=0.001). Being moderately food insecure more than doubled the odds of dropout (OR=2.50; 95% CI: 1.12-5.55, p=0.025). Severe mental distress increased the likelihood of dropout more than seven-fold (OR=7.08; 95% CI: 2.67-18.81, p<0.001).

**Table 4:** Population-weighted multivariate logistic correlates to dropout among study participants.

	OR (95% CI)	Standard error	p-value
Sex			
Female	ref		
Male	2.70 (1.48- 4.89)	0.82	0.001*
Financial-aid recipient			
No	ref		
Yes	0.59 (0.31-1.14)	0.20	0.117
Residence during lockdown situated in:			
City/Suburb	ref		
Township	0.64 (0.29-1.45)	0.27	0.288
Town	0.93 (0.27-3.24)	0.59	0.913
Village/farm	2.14 (0.99, 4.63)	0.84	0.053
Self- reported COVID-19 infection (of participant and/or close friends and family)			
No	ref		
Yes	2.10 (0.83-5.36)	1.00	0.119
Working from home challenging			
No			
Yes	0.64 (0.35-1.17)	0.20	0.147
Food insecurity			
Food secure	ref		
Mildly food insecure	0.98 (0.36- 2.68)	0.50	0.975
Moderately food insecure	2.50 (1.12- 5.55)	1.02	0.025*
Severely food insecure	1.09 (0.46- 2.58)	0.48	0.838
Mental distress			
Minimal	ref		
Mild	1.14 (0.41- 3.22)	0.60	0.802
Moderate	1.39 (0.48- 4.01)	0.75	0.546
Severe	7.08 (2.67-18.81)	3.53	<0.001*

*Significance at p<0.05.

## 4 Discussion

Student dropout is a key challenge faced by higher education institutions worldwide (4). Literature affirms that the determinants of dropout are intricate and greatly influenced by context (6). The COVID-19 pandemic added an additional complexity, as evidenced by the findings of various studies across the world acknowledging its contribution to increased dropout rates (1, 9). The current study found a dropout rate of 10.5% (95% CI: 8.2-13.2) during the COVID-19 pandemic, a slight increase from the 10% dropout rate cited in pre-COVID-19 research at the same institution of the current work (20). It also found that being a male student and being older was significantly linked with dropout, a finding aligned with literature (4, 44). However, closer inspection of the current work reveals that students residing in villages or farms during the lockdown had dropout rates of closer to 20%, nearly double the average, pre-pandemic rate. Literature emphasizes that, beyond the well-documented factors impacting dropout rates, students in remote areas encountered additional challenges in remote learning during the COVID-19 pandemic compared to their counterparts in different regions. These challenges stemmed from unreliable internet connections and unpredictable power supply leading to class absences, an important precursor of dropout (4, 45).

Further to this, the current study reported high levels of severe food insecurity (25.7%; 95% CI: 22.3-29.4) and severe mental distress (26.7%; 95% CI: 23.3-30.4), together with evidence that moderate food insecurity (OR=2.50; 95% CI: 1.12-5.55, p=0.025) as well severe mental distress (OR=7.08; 95% CI: 2.67-18.81, p<0.001) significantly increased the likelihood of student dropout. Again, it was students residing in villages or on farms during the pandemic that were most affected, with 43.6% of these students reporting severe food insecurity. It is also important to highlight that those students with moderate food insecurity had the highest rate of dropout at 17.5% (p=0.046). It is possible that these students may not have qualified for social support programs, as priority is often given to students who are severely food insecure and those grappling with hunger (46). The elevated levels of severe food insecurity and mental distress may reinforce poor classroom participation due to impaired concentration and reduced cognitive functioning, both impacting negatively on the learning experience and reflected in dropout rates (47, 48).

The data presented suggests an intriguing link between food insecurity, mental distress, and dropout rates amid the COVID-19 pandemic. Both food insecurity and mental distress have been demonstrated to affect academic outcomes directly as well as through interactions with each other (27, 49, 50). However, the current findings demonstrate how these dynamics may have evolved during the COVID-19 pandemic, which fostered isolation rather than the integration advocated for by Tinto. While noting the multifactorial nature of student dropout, it is plausible that the challenges some students faced with remote learning could have contributed to their dropout. Furthermore, the heightened risk of food insecurity and mental distress because of challenges including mass job loss and isolation due to the pandemic, may have also had a potential impact on dropout. These findings are aligned with work from South America which found that 38% of students reported economic distress (related to food insecurity risk) as a key motive to dropout early in the pandemic, while dropout motives related to mental distress increased over time reaching 40% in 2021 (1). There is also evidence linking poor family resources as well as an inability to cope with low resilience, and ultimately heighted risk for dropout, further corroborating the findings from the current study (51).

Tinto’s theory highlights the crucial role of student integration into the social and academic aspects of university life, arguing that successful integration reduces the likelihood of dropout. The current study takes into consideration the consequences of the COVID-19 pandemic on academic activities, recognizing its impact on student learning, as well as the pandemic’s effects on socio-economic conditions, including food insecurity, and mental distress risk (11, 13, 16). These impacts have negative consequences on the social and academic integration that Tinto highlights as important. Remote learning, exacerbated by issues including inadequate connectivity and intermittent power supply as reported in the literature, emerges as a substantial hindrance to the integration between students and the academic environment. Our research embraces Tinto’s work by highlighting that food insecurity and mental distress also perpetuate social isolation arising from the mental toll of physical distancing and economic devastation of the pandemic, thereby leading to higher levels of dropout. This work therefore identifies these factors as important contributors to the complexity of students’ experiences and decision-making processes during the pandemic. This research suggests that food insecurity and mental distress, during the COVID-19 pandemic, intersect with Tinto’s core concept of integration. It enriches our understanding of the nuanced dynamics and interplay between these factors and student dropout.

### 4.1 Strengths and limitations

The current study has several strengths. First, a study of this nature, that aimed to investigate the relationship between student dropout, food insecurity and mental distress during the COVID-19 pandemic, has not previously been conducted in South Africa- in part, due to the difficulty of following up a cohort of students to assess student dropout rates. Second, a weighting was applied to our survey sample to ensure representation of the student population by sex and population group. Third, the survey sample was diverse in terms of race, representative of the South African higher education sector. However, the study has also some limitations. Given the complexity of the COVID-19 pandemic and its both direct and indirect, realised and unknown consequences on individuals, the observed prevalence and interactions between student dropout, food insecurity and mental distress cannot be solely attributed to the COVID-19 pandemic. Furthermore, student dropout does not account for students who persist in other higher education institutions. In addition to this, findings are from one university in South Africa, and therefore cannot be generalized to the Republic as a whole. Finally, self-selected sampling or self-reporting may have created bias. To address the potential selection bias, the authors have weighted the findings to the underlying student population.

### 4.2 Practical implications

The global higher education landscape experienced significant upheaval due to the COVID-19 pandemic. There is a growing perspective suggesting the likelihood of more severe pandemics in the future. Regardless, disruptions can manifest in various other ways, such as violent student protests, a frequent occurrence in South Africa, economic downturns, and political instability, all of which have the potential to adversely affect groups of university students. These disruptions are likely to have repercussions on economic aspects, including food insecurity, and the mental well-being of university students. Furthermore, in part due to the COVID-19 pandemic, new ways of delivering higher education, including hybrid models of delivery are being explored. In light of the present study, it will be important to consider psychosocial and mental health factors when designing these models. Identifying ways of allowing students to potentially learn remotely yet maintain a sense of inclusivity and connectedness as well as ensuring food security will likely contribute to reduced dropout rates.

The present study recognizes these factors, along with other critical elements, as crucial contributors to students’ decisions to dropout. Collectively, this awareness presents an opportunity for higher education institutions to take a proactive approach in implementing strategies to retain students during periods of disruption and implementation of innovative, hybrid teaching methods, by targeting the food security and mental well-being of its student population.

## 5 Conclusion

University dropout rates remain a concern in higher education, with levels from one South African university found to slightly increase during the recent COVID-19 pandemic. Food insecurity and severe mental distress, two factors heavily impacted by the COVID-19 pandemic, were found to be strong predictors of student dropout with future studies needed to explore whether the changing trends identified in the current work persist after the COVID-19 pandemic. Regardless, given the known impact of food insecurity and mental distress on student success, institutions of higher education should provide targeted support to students found to be food insecure and those who are experiencing mental distress, thereby improving student success and reducing dropout.

## Data availability statement

The raw data supporting the conclusions of this article will be made available by the authors, without undue reservation.

## Ethics statement

The studies involving humans were approved by University Human Research Ethics Committee (Non-medical) (H20/06/22) University of the Witwatersrand Human Research Ethics Committee (HREC) (Medical) (M210712). The studies were conducted in accordance with the local legislation and institutional requirements. The participants provided their written informed consent to participate in this study.

## Author contributions

FW: Conceptualization, Data curation, Formal analysis, Investigation, Methodology, Writing – original draft, Funding acquisition, Writing – review & editing. RW: Conceptualization, Writing – review & editing, Methodology. LM: Investigation, Writing – review & editing, Project administration. MM: Conceptualization, Investigation, Writing – review & editing, Funding acquisition, Methodology, Project administration. UK: Supervision, Writing – review & editing. FG: Supervision, Writing – review & editing.
